# Genomic and Proteomic Analyses of Extracellular Products Reveal Major Virulence Factors Likely Accounting for Differences in Pathogenicity to Bivalves between *Vibrio mediterranei* Strains

**DOI:** 10.3390/ani14050692

**Published:** 2024-02-22

**Authors:** Congling Fan, Wenfang Dai, Haiyan Zhang, Sheng Liu, Zhihua Lin, Qinggang Xue

**Affiliations:** 1 Zhejiang Key Laboratory of Aquatic Germplasm Resource, College of Biological Environmental Sciences, Zhejiang Wanli University, Ningbo 315100, China; fancongling1@126.com (C.F.); daiwenfang6283@163.com (W.D.); haiyan.zhang@zwu.edu.cn (H.Z.); liusheng3729@163.com (S.L.); zhihua9988@126.com (Z.L.); 2 Ninghai Institute of Mariculture Breeding and Seed Industry, Zhejiang Wanli University, Ninghai, Ningbo 315604, China

**Keywords:** *Vibrio mediterranei*, extracellular products, virulence factor, pathogenicity

## Abstract

**Simple Summary:**

The extracellular products (ECPs) secreted by virulent *Vibrio mediterranei* strains are pathogenic to bivalves. However, it remains unclear whether differences in pathogenicity between different *V. mediterranei* strains are associated with ECP compositions. In this study, genomics and proteomics approaches were integrated to compare the virulence factors in the ECP proteins secreted by two high-virulence *V. mediterranei* strains and one low-virulence strain. We found that the ECPs secreted by high-virulence *V. mediterranei* strains had a greater number and variety of virulence factors compared to low-virulence strains, and that 73 of the 95 specific ECP proteins in the high-virulence strains were not expressed in the low-virulence strain. These findings provide further insights into the role that virulent ECPs play in the pathogenesis of *V. mediterranei* to bivalves.

**Abstract:**

*Vibrio mediterranei*, a bacterial pathogen of bivalves, has exhibited strain-dependent virulence. The mechanisms behind the variations in bivalve pathogenicity between *V. mediterranei* strains have remained unclear. However, a preliminary analysis of the extracellular product (ECP) proteomes has revealed differences in protein compositions between low- and high-virulence strains; in addition to 1265 shared proteins, 127 proteins have been identified to be specific to one low-virulence strain and 95 proteins to be specific to two high-virulence strains. We further studied the ECP proteins of the three *V. mediterranei* strains from functional perspectives using integrated genomics and proteomics approaches. The results showed that lipid metabolism, transporter activity and membrane transporter pathways were more enriched in the ECPs of the two high-virulence strains than in those of the low-virulence strain. Additionally, 73 of the 95 high-virulence strain-specific proteins were found to have coding genes in the genome but were not expressed in the low-virulence strain. Moreover, comparisons with known virulence factors in the Virulence Factor Database (VFDB) and the Pathogen–Host Interactions Database (PHI-base) allowed us to predict more than 10 virulence factors in the categories of antimicrobial activity/competitive advantage, the effector delivery system and immune modulation, and the high-virulence strain-specific ECP proteins consisted of a greater percentage of known virulence factors than the low-virulence strain. Particularly, two virulence factors, MtrC and KatG, were identified in the ECPs of the two high-virulence strains but not in those of the low-virulence strain. Most coding genes of the ECP proteins including known virulence factors were identified on chromosome 1 of *V. mediterranei*. Our findings indicate that variations in virulence factor composition in the bacterial ECPs may partially account for the differences in the bivalve pathogenicity between *V. mediterranei* strains.

## 1. Introduction

*Vibrios* are rod-shaped, Gram-negative bacteria naturally present in freshwater, estuarine and marine environments [1,2]. While most *Vibrio* species constitute a normal component of the microbial ecology in the related environments, some species are causative agents of vibriosis in plants, animals and humans [3,4]. At least 10 *Vibrio* species, for example, have been reported to be pathogenic or opportunistically pathogenic to humans [4,5].

*Vibrios* also encompass important pathogens of aquatic vertebrates and invertebrate animals, especially bivalves [6]. *Vibrios* infection causes larval vibriosis that represents a major threat to hatchery-reared larvae and spats of several bivalves [7,8,9,10,11]. In 2007, a *Vibrio tubiashii*-caused vibriosis resulted in an 80% mortality, leading to a 59% production reduction in oyster larvae in hatcheries on the West Coast of North America [8]. *Vibrio* infections also give rise to mortalities in adult bivalves. *Vibrio alginolyticus*, for example, was identified as the causative agent for a summer mortality incident in cultured Pacific oysters *Crassostrea gigas* in China [12]. Other *Vibrio* species that are reported to cause vibriosis in bivalves and other aquatic animals include *Vibrio mediterranei* [13], *Vibrio carchariae* [14], *Vibrio splendidus* [15], *Vibrio aestuarianus* [16], *Vibrio neptunius* [10], *Vibrio anguillarum* [17], *Vibrio coralliilyticus* [18] and *Vibrio parahaemolyticus* [19].

However, pathogenicity is not a species-specific quality for these pathogenic *Vibrios*. Significant differences in the virulence and the degree of pathogenicity are observed between strains of the above-mentioned pathogenic or opportunistically pathogenic *Vibrio* species [20,21]. This difference in virulence between strains within a group or species is determined by the qualitative or quantitative variations in virulence factors [22]. Virulence factors are defined as pathogen components that damage the host [23,24]. Bacterial virulence factors consist of extremely diverse components that are involved in the entire host infection process including adherence, invasion and survival in the host and environments. The latest version of the Virulence Factor Database (VFDB), for example, classifies 11,027 bacterial virulence factors into 14 categories (accessed on November 2023) [25]. A wide variety of virulence factors associated with the adherence, motility, invasion, iron uptake subcategory of the nutritional/metabolic factor, exotoxin, effector delivery system, exoenzyme, regulation and biofilm have been identified in *Vibrio* species pathogenic to aquatic animals [12,26,27,28,29,30].

Pathogenic bacteria secrete virulence factors as compositions of extracellular products (ECPs), which are believed to play a role in the bacteria’s pathogenicity. Aquatic pathogen ECPs have been reported to contain proteases, lipases, DNases, chitinases, hemolysins and polysaccharides [31,32,33]. These factors contribute to extensive tissue damage, enabling bacteria to acquire nutrients and proliferate within hosts [33,34]. Certain components, such as ECPs, serine proteases, and metalloproteases, have been documented as lethal to shrimp and oyster larvae [35,36]. Haemolysins are also implicated in the pathogenicity of bacteria toward shrimp and fish [37,38]. Additionally, ECP chitinases play a role in facilitating bacterial penetration of crustacean shells [39]. However, it remains to be further studied whether the qualitative and/or quantitative differences in ECP virulence factors represent a determinant factor for the intra-specific variations in the pathogenicity of a bacterial pathogen.

We have characterized *V. mediterranei* as the causative agent of a recent massive mortality incident in a razor clam hatchery [13]. Differences in pathogenicity to larval and juvenile razor clams and oysters have been observed between bacterial strains in experimental infections with cultured bacteria [13]. ECPs of one low-virulence strain (DT02) and two high-virulence strains (DT07 and RW01) also demonstrate correspondent virulence differences [13]. In addition, DT02 ECPs contain higher metalloproteinase and chitinase activities than the DT07 and RW01 ECPs [13]. Intriguingly, proteomic analyses of the ECPs of the three *V. mediterranei* strains using liquid chromatography–tandem mass spectrometry (LC-MS/MS) revealed 1265 proteins shared by all three strains, 95 ECP proteins specific to the two high-virulence strains (i.e., DT07 and RW01), and 127 ECP proteins specific to the one low-virulence strain (i.e., DT02) [13]. However, the functional significance of the differences in the proteome composition between *V. mediterranei* strains with different virulence remained to be investigated.

In the present research, we performed a comprehensive function analysis of the ECP proteins identified in the previous research [13] with combined genomics and proteomics approaches. Our goals were to: (1) functionally annotate the 1265 proteins shared by all three strains, the 95 proteins specific to the two high-virulence strains and the 127 proteins specific to the one low-virulence strain using KEGG pathway and GO term analyses; (2) identify known virulence factors in the ECP proteins through searching published virulence factor databases; (3) determine the coding genes of the ECP proteins in the bacterial genomes; and (4) assess the expression of the related coding genes in different *V. mediterranei* strains. The results may provide important information about the mechanisms underlying the intra-specific virulence differences in *V. mediterranei* and other bacterial pathogens.

## 2. Materials and Methods

### 2.1. V. mediterranei Strains, Proteomic Sequence Datasets and Genomic Sequences

*V. mediterranei* strain isolation and characterization, ECP preparation and proteomics analysis were reported in detail previously [13]. Bacterial strain DT02, a low-virulence strain isolated from a diseased razor clam juvenile, and strains DT07 and RW01, 2 high-virulence strains isolated from a diseased razor clam juvenile and rearing water, respectively, were used in this research. The proteomics datasets selected for analysis included the 1265 ECP proteins shared by all three bacterial strains, the 95 ECP proteins specific to the two high-virulence strains and the 127 ECP proteins specific to the one low-virulence strain.

Genome sequencing of the three *V. mediterranei* strains was carried out using high-throughput sequencing in the Illumina NovaSeq 6000 system and nanopore sequencing in an Oxford Nanopore PromethION 48 platform. The genomes consisted of 2 circular chromosomes of 3,762,826 bp and 2,025,510 bp, respectively, and 10 plasmids for strain DT02, 2 circular chromosomes of 3,666,428 bp and 2,211,640 bp, respectively, 1 integron and 5 plasmids for strain DT07, and 2 circular chromosomes of 3,771,825 bp and 2,182,795 bp, respectively, and 6 plasmids for strain RW01. The bacterial genomes were de novo assembled using SMRT Link v5.1.0 (https://www.pacb.com/support/software-downloads/), (accessed on 9 November 2021) and Unicycle (https://github.com/rrwick/Unicycler), (accessed on 9 November 2021), and further optimized to correct the misassembled regions using arrow software v2.2.1 [40,41]. Protein-coding genes were predicted using the program Glimmer 3 [42]. Predicted coding gene sequences were compared with bacterial proteins in the UniProt database using the program BLASTX [43]. The average nucleotide identity (ANI) of the genomes between strains was determined using the program ANItools (http://ani.mypathogen.cn/), (accessed on 13 June 2023) [44].

### 2.2. Identification of ECP Protein-Coding Genes in the Genomes

Coding genes of the ECP proteins determined by proteomics were identified by aligning the amino acid sequence to the genome sequences of the three *V. mediterranei* strains using the program BLAST GUI Wrapper in TBtools [45]. The number of genes distributed on each chromosome was plotted as a histogram using Chiplot Tools (https://www.chiplot.online/), (accessed on 24 July 2023).

### 2.3. Functional Annotations of the ECP Proteins

General biological functions of the *V. mediterranei* ECP proteins were predicted using Gene Ontology (GO) enrichment analysis and Kyoto Encyclopedia of Genes and Genomes (KEGG) pathway analysis [46,47]. GO analyses were carried out using the Gene Ontology Annotation (GOA) database at the UniProt Knowledgebase (UniProtKB) (http://www.ebi.ac.uk/GOA/), (accessed on 22 July 2023). KEGG pathway analyses were done first by getting each protein’s KEGG annotation using the KEGG Automatic Annotation Server (KAAS) (http://www.genome.jp/kaas-bin/kaas_main), (accessed on 21 July 2023) and then by mapping the annotated proteins into KEGG pathways using the KEGG Mapper online (http://www.kegg.jp/kegg/mapper.html), (accessed on 21 July 2023). GO enrichment and KEGG pathway analysis results were compared between protein sets and plotted in histograms using the OmicShare Tools (https://www.omicshare.com/tools), (accessed on 23 July 2023).

### 2.4. Prediction of Virulence Factors

Bacterial virulence factors were predicted by online searching the Virulence Factor Database (VFDB) (www.mgc.ac.cn/VFs/main.htm), (accessed on 9 October 2023) [48] and the Pathogen–Host Interactions Database (PHI-base) (www.phi-base.org/), (accessed on 12 October 2023) [49]. VFDB categorizes all reported bacterial virulence factors according to their functions in the pathogenesis process, and PHI-base uses pathogen–host interactions as virulence attributes and classifies them into 9 high-level phenotypes according to the interaction outcomes [25,50].

### 2.5. Quantitation of ECP Protein Expression

Protein expression was assessed based on the peak intensity of each protein in the LC-MS/MS detection. Peak intensities of each protein were normalized in a globally fashion [51,52]. The normalized values were then treated as protein expression levels, and the results were presented in Heatmap.

## 3. Results

### 3.1. Coding Genes of the ECP Proteins

For the 1265 ECP proteins shared by all three strains, a total of 1165 coding genes were identified in the three genomes, but each genome was determined to host different numbers of coding genes, with 1154 in DT07, 1153 in RW01 and 1151 in DT02. Differences in specific genes contained in each genome were also observed. The DT07 genome contained 9 and 12 genes that were not identified in RW01 and DT02, respectively. These DT07-specific coding genes included known virulence factors (*plr*/*gapA*, *purCD*, *plr*/*gapA*, *kasB* and *nmpC*). In the meantime, the DT07 genome lacked eight genes contained in the RW01 genome and eight genes in the DT02 genome (Table 1). One hundred of the shared ECP proteins were not identified to have coding genes in any of the three genomes (Table S1).

For the 95 ECP proteins specific to the two high-virulence strains, 72 coding genes were determined in the DT07 genome and 73 in the RW01 genome. The low-virulence DT02 genome was also found to contain 66 coding genes for the 95 ECP proteins specific to the two high-virulence strains. The DT02 genome lacked seven genes, including the coding genes for the virulence factor *katG*, compared to the two high-virulence strains. In addition, the RW01 genome contained a coding gene for the virulence factor *tcdA*, which was not found in the DT07 genome (Table 2). A total of 22 proteins did not have coding genes in any of the three genomes (Table S2).

For the 127 ECP proteins specific to the low-virulence strain DT02, 91 coding genes were determined and 28 genes were missing in the strain’s genome (Table S3). The genomes of the two high-virulence strains contained 92 coding genes. The DT02 genome contained seven genes that were not identified in the DT07 genome and six genes that were missing in the RW01 genome, but it lacked eight genes that were present in the DT07 genome and seven genes that were absent in the RW01 genome (Table 3).

### 3.2. Functional Annotation and Pathway Assignment of ECP Proteins

**Figure 1:**
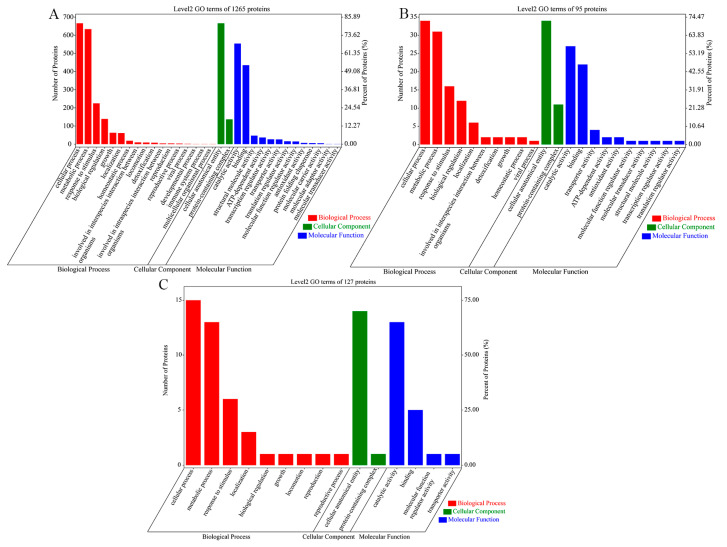
GO analysis of the 3 sets of *V. mediterranei* ECP proteins. The numbers of ECP proteins and their percentages among the proteins analyzed are shown. (**A**) The 1265 ECP proteins shared by all 3 strains; (**B**) 95 proteins specific to the 2 high-virulence strains; (**C**) 127 proteins specific to the 1 low-virulence strain.

GO enrichment analysis annotates proteins in GO terms and categorizes them in three aspects, biological process, cellular component and molecular function. For all three sets of *V. mediterranei* ECP proteins, the first two most-enriched GO terms were the cellular process and the metabolic process in the biological process category, the cellular anatomical entity and the protein-containing complex in the cellular component category, and catalytic activity and binding in the molecular function category (Figure 1). Compared to the 1265 ECP proteins shared by all three strains and 127 proteins specific to the one low-virulence strain, however, the 95 proteins specific to the two high-virulence strains showed a relatively higher percentage enrichment in the GO terms of response to stimulus, biological regulation, homeostatic process, interspecies interaction between organisms, detoxification, viral process, protein-containing complex, transporter activity, antioxidant activity and molecular transducer activity.

**Figure 2:**
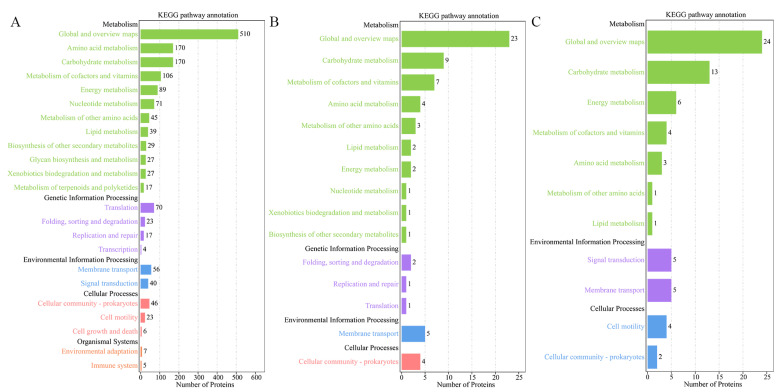
KEGG analysis of the 3 sets of *V. mediterranei* ECP proteins. (**A**) The 1265 ECP proteins shared by all 3 strains; (**B**) 95 proteins specific to the 2 high-virulence strains; (**C**) 127 proteins specific to the 1 low-virulence strain.

KEGG pathway analysis functionally annotates proteins into different pathways that are further classified in categories of metabolism, genetic information processing, environment information processing, cellular process and organismal systems. For all three *V. mediterranei* ECP protein sets, the most annotated pathways were in the metabolism category, with global and overview maps and carbohydrate metabolism being the most abundant (Figure 2). The 1265 ECP proteins shared by all three strains involved five categories with 23 pathways (Figure 2A). The 95 proteins specific to the two high-virulence strains and the 127 proteins specific to the one low-virulence strain were identified to have significantly fewer pathways than the 1265 shared proteins, and particularly, neither involved the organismal systems category (Figure 2B,C). Compared to the three strains’ shared proteins and the low-virulence strain-specific proteins, the 95 proteins specific to the two high-virulence strains showed higher relative abundance for the pathways of metabolism of cofactors and vitamins, metabolism of other amino acids, lipid metabolism, folding, sorting and degradation, replication and repair, membrane transport, and cellular community-prokaryotes (Table 4).

### 3.3. Virulence Factors in ECP Proteins

**Figure 3:**
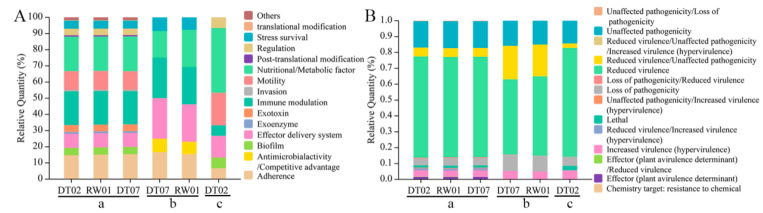
Percentage of virulence factor classes in the 3 sets of *V. mediterranei* ECP proteins identified by searches of the databases VFDB (**A**) and PHI-base (**B**). a: the 1265 ECP proteins shared by all 3 strains; b: 95 proteins specific to the 2 high-virulence strains; c: 127 proteins specific to the 1 low-virulence strain.

Virulence factors in each of the three sets of *V. mediterranei* ECP proteins were predicted by comparison of known virulence factors via searches of the databases VFDB and PHI-base (Figure 3). VFDB searches identified 246 virulence factors belonging to 13 classes in the 1265 ECP proteins shared by all three strains, and most of them involved the nutritional/metabolic factor, immune modulation, adherence and motility (Figure 3A and Table S4). The searches also identified 13 virulence factors in the 95 ECP proteins specific to the two high-virulence strains, which belonged to six classes, and most of them involved the nutritional/metabolic factor, immune modulation, the effector delivery system and adherence (Figure 3A and Table S5). In the 127 proteins specific to the one low-virulence strain, VFDB searches identified 15 virulence factors that belonged to seven classes, and most of them involved the nutritional/metabolic factor, motility and the effector delivery system (Figure 3A and Table S6). The virulence factors that were related to antimicrobial activity/competitive advantage, the effector delivery system and immune modulation exhibited a higher percentage in the 95 proteins specific to the two high-virulence strains than in the 1265 ECP proteins shared by all three strains and 127 proteins specific to the one low-virulence strain (Figure 3A). In addition, the high-virulence ECP proteins contained a membrane fusion protein, MtrC, that was not identified in the two ECP protein sets (Table S5).

The PHI-base search predicted 583 virulence factors that were categorized into seven classes in the 1265 ECP proteins shared by all three strains, and most of them were related to the classes of reduced virulence, unaffected pathogenicity/loss of pathogenicity, increased virulence (hypervirulence) and reduced virulence/unaffected pathogenicity (Figure 3B and Table S7). The PHI-base search identified 20 virulence factors that belonged to four classes, and most of them were associated with the classes of reduced virulence and reduced virulence/unaffected pathogenicity (Figure 3B and Table S8). In the 127 proteins specific to the one low-virulence strain, the PHI-base search found 35 virulence factors that involved five classes, and most of them belonged to the classes of reduced virulence, unaffected pathogenicity and loss of pathogenicity (Figure 3B and Table S9).

### 3.4. Distributions of ECP Protein-Coding Genes in Genomes

**Figure 4:**
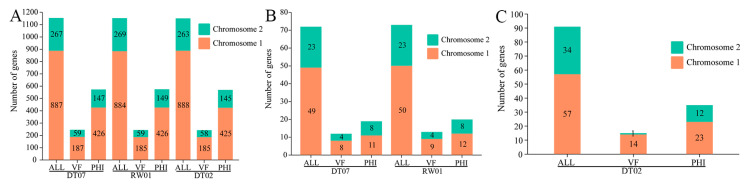
Distributions of the coding genes for the ECP proteins shared by all 3 *V. mediterranei* (**A**), specific to the high-virulence strains (**B**) and the low-virulence strain (**C**). ALL: all ECP protein-coding genes in the strain; VF: virulence factors in ECP proteins searched in VFDB; PHI: virulence factors in ECP proteins searched in PHI-base.

Analysis of the genomes of the three *V. mediterranei* strains disclosed that the coding genes for the ECP proteins identified in this study, including the predicted virulence factors, were present on the two chromosomes. No ECP protein-coding genes were found in non-chromosome genome elements. In addition, chromosome 1 contained most of the ECP protein-coding genes (Figure 4).

### 3.5. Expression of Genes Coding for ECP Proteins

**Figure 5:**
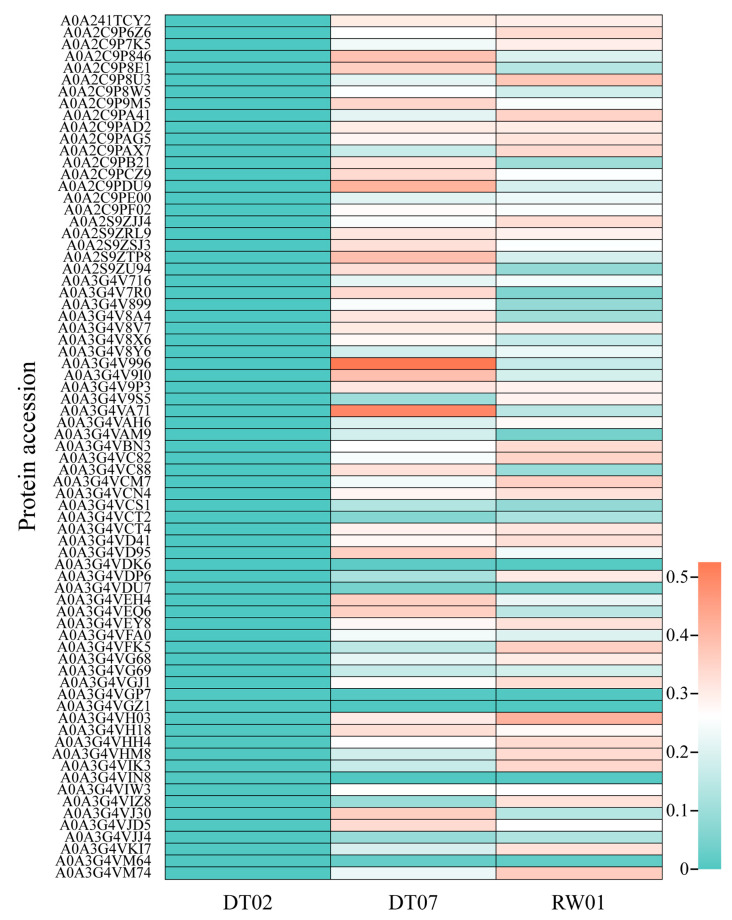
Expression of 73 out of 95 ECP proteins specific to the 2 high-virulence strains with coding genes in the 3 *V. mediterranei* strains.

The coding genes of 1165 out of 1265 ECP proteins, which are shared by all three strains, were found to be expressed in all strains. However, among the 95 ECP proteins specific to the two high-virulence strains, 73 had coding genes present in three genomes and were expressed only in the two high-virulence strains. Additionally, variations in the expression levels of coding genes were observed between the two high-virulence strains. Specifically, 17 coding genes exhibited higher expression levels in DT07 compared to RW01, while 13 coding genes showed higher expression levels in RW01 compared to DT07 (Figure 5).

## 4. Discussion

ECPs secreted by *Vibrios* are associated with the bacteria’s pathogenicity to bivalves [13,16,53]. Some components such as proteases are believed to play a role in this attribute of bivalve-pathogenic *Vibrios* [16,54]. We have characterized *V. mediterranei* as the causative agent of larval vibriosis in hatchery-reared clams and oysters, and uncovered variations in pathogenicity between the bacterial isolates. Additionally, the differences in enzyme activities and in the proteomic compositions of the ECPs from bacterial strains differing in virulence also suggested the possible association with the bacterium’s pathogenicity [13]. Results of a combined genomic and proteomic analysis and particularly the discovery of quantitative and qualitative variations in virulence factors in ECPs provide further insights into the mechanism underlying the intra-specific variations in bivalve pathogenicity of *V. mediterranei* in this research.

Our analyses revealed the differences in the general proteomic composition and virulence factors between ECPs from different *V. mediterranei* strains, indicating the contribution of the bacterial ECPs to the bacterial virulence. At the proteomic level, both low- and high-virulence strains were observed to have proteins in their ECPs [13]. Annotation of the shared and strain-specific proteins further suggests their functional differences. We found in the GO enrichment and KEGG pathway analyses that the pathways involved in lipid metabolism and transporter activity and the membrane transporter pathways are more enriched in the 95 high-virulence strain-specific proteins as compared to those in the 1265 shared and 127 low-virulence-specific proteins. The lipid metabolism pathway is thought to be important for pathogenic bacteria to establish stable infections in hosts [55]. In addition, the membrane transporter KEGG pathway and the transporter activity GO term are associated with increased virulence in pathogenic bacteria [56].

More importantly, differences in the ECPs containing known virulence factors were found between the two types of bacterial strains. For example, 13.7% (13/95) of the high-virulence strain-specific ECP proteins are predicted to be virulence factors. This ratio was 11.8% (15/127) for the low-virulence strain. It appears that the high-virulence ECPs contain more virulence factors that are involved in pathogen adherence, the effector delivery system and host immune modulation. Several predicted virulence factors that are present in the ECPs of high-virulence *V. mediterranei* strains but not in the low-virulence strain are particularly worth noting. These include the stress survival-related virulence factor KatG, the antimicrobial activity/competitive advantage-related virulence factor MtrC, and the unaffected pathogenicity-related virulence factor ToxR. KatG is a catalase that degrades hydrogen peroxide and organic peroxides, and has been reported to protect pathogenic bacteria from the toxic effects of organic peroxides and enhance drug resistance, and thereby facilitate their survival and growth in hosts [57,58,59,60]. MtrC is a component of the MtrCDE efflux system [61]. The system is found to protect pathogenic bacteria from antimicrobial substances on mucosal surfaces and promotes their survival during infection [62]. ToxR is a transmembrane transcriptional regulator [63,64]. *V. harveyi* strains with higher *toxR* expression levels exhibit higher virulence to shrimp [65]. Thus, these high-virulence-specific virulence factors likely play a part in the increased pathogenicity in the related *V. mediterranei* strains. This phenomenon has been observed in other pathogenic *Vibrios*; the virulence factor *pJM1* is important to the pathogenicity of the high-virulence *V. anguillarum* strain [66]. It should also be noted that there were differences in virulence factor composition between the two high-virulence *V. mediterranei* strains in this research. The virulence factors *plr*/*gapA* and *purCD* contained in strain DT07 were not found in strain RW01. A comparable observation has been made in the human pathogen *Vibrio cholerae*, wherein virulence factors such as *ctxA*, *ctxB*, and *tcpA*, present in the pathogenic non-O1 strain, are notably absent in the pathogenic O1 strain [67]. These findings suggest that virulence factors may function differently in different pathogenic bacteria.

Genomic analysis determined that the coding genes of all the ECP proteins from the three *V. mediterranei* strains, including those for the virulence factors, are present in the two chromosomal genomes. It is speculated that genes in the chromosomes are usually related to the phenotypes at the species level of a bacterium [68]. In contrast, some strain-specific phenotypes such as antibiotic resistance and virulence factors are often controlled by genes carried by plasmids [69]. This appears to be inconsistent with our results regarding the distribution of ECP protein-coding genes in the bacterial genomes. One explanation for the phenomenon may be related to the regulation at the gene expression level. Indeed, we found that most (66/95) of the coding genes for the high-virulence strain-specific ECP proteins were also present in the genome of the one low-virulence strain, which is, in fact, close to the number of coding genes identified in the genomes of the two high-virulence strains (72 and 73). It is apparent that the expression of those related genes in the low-virulence genome is inhibited by certain regulators. The mechanisms underlying the regulation of gene expression should be approached in future studies. On the other hand, differences in the number of coding genes and the specific genes involved in the different strains may involve the species evolution of *V. mediterranei,* and the associated mechanisms also need to be further studied. Interestingly, the coding genes for some ECP proteins identified in the proteomic analysis are found in the corresponding genomes. Studies will be carried out to investigate whether this is related to technical errors, unidentified genes in the genomes or environmental contaminations.

## 5. Conclusions

To summarize, a comprehensive analysis of the ECP protein compositions of three *V. mediterranei* strains that vary in pathogenicity to bivalves using a combination of genomics and proteomics resulted in the discovery of functional variations between low- and high-virulence strains. Particularly, differences in virulence factor composition in ECPs are observed. These findings provide solid evidence for the speculation that bacterial ECPs play an important role in the previously observed virulence differences between *V. mediterranei* strains. In addition, the results of our present research suggest that the inter-strain variations of ECP composition, including the virulence factors, are likely controlled by the regulation of expression of related coding genes. Future studies on the evolution and regulation of expression of the ECP protein-coding genes will generate more information about the pathogenesis of *V. mediterranei* and other Vibrio species in aquatic organisms.

## Figures and Tables

**Table 1:** Information for 30 ECP proteins with different coding genes for the 1265 ECP proteins shared by all 3 strains.

Protein ID	Carrier Strain	Gene Name	Gene ID	Protein Function	VFDB ID
A0A241TBU8	RW01	*ECB94_2627*	-	NUDIX hydrolase	-
A0A241TD21	DT07/RW01	*ECB94_2193*	-	Transcriptional regulator	-
A0A2C9P8E4	DT07/DT02	*dapB*	-	4-Hydroxy-tetrahydrodipicolinate reductase	-
A0A2C9P8Q5	DT07	*C998_17345*	-	Glyceraldehyde-3-phosphate dehydrogenase	*plr*/*gapA*
A0A2C9PAH6	RW01	*atpF*	*atpF*	ATP synthase subunit b	-
A0A2C9PAJ3	DT07/DT02	*orn*	-	Oligoribonuclease	-
A0A2C9PCL2	DT07/RW01	*fabB*	*fabB*	Beta-ketoacyl-ACP synthase I	*kasB*
A0A2S9ZJ94	DT07/RW01	*rplC*	-	5S ribosomal protein L3	-
A0A2S9ZN28	RW01/DT02	*ampD*	-	1,6-Anhydro-N-acetylmuramyl-L-alanine amidase AmpD	-
A0A2S9ZRI4	DT07	*C998_9425*	-	MarR family transcriptional regulator	-
A0A3G4V556	RW01/DT02	*ECB94_66*	-	UPF234 protein ECB94_66	-
A0A3G4V5Q8	DT07/DT02	*purC*	-	Phosphoribosylaminoimidazole-succinocarboxamide synthase	*purCD*
A0A3G4V5Z8	RW01/DT02	*C998_16755*	-	Probable phosphatase ECB94_197	-
A0A3G4V6B1	DT07/RW01	*ECB94_262*	-	Aminotransferase	-
A0A3G4V6Y7	RW01/DT02	*ackA*	-	Acetate kinase	-
A0A3G4V9F9	DT02	*ECB94_8*	-	Putrescine-binding periplasmic protein	-
A0A3G4VA61	DT07/DT02	*rplE*	*rplE*	5S ribosomal protein L5	-
A0A3G4VB02	DT07/DT02	*surA*	-	Chaperone SurA	-
A0A3G4VB66	DT07/RW01	*proS*	*proS*	Proline–tRNA ligase	-
A0A3G4VBI1	RW01/DT02	*treC*	-	Alpha, alpha-phosphotrehalase	-
A0A3G4VBT8	DT07/DT02	*alaS*	-	Alanine–tRNA ligase	-
A0A3G4VCV5	DT02	*ECB94_15585*	-	Threonylcarbamoyl-AMP synthase	-
A0A3G4VF14	DT07/DT02	*menB*	-	1,4-Dihydroxy-2-naphthoyl-CoA synthase	-
A0A3G4VF71	DT07/RW01	*ECB94_147*	-	Dipeptide epimerase	-
A0A3G4VH60	DT07/RW01	*mtlD*	-	Mannitol-1-phosphate 5-dehydrogenase	-
A0A3G4VHP1	DT07/RW01	*tkt*	-	Transketolase	-
A0A3G4VHT5	RW01/DT02	*ECB94_24335*	-	IclR family transcriptional regulator	-
A0A3G4VJM1	DT02	*ECB94_25375*	-	UPF52 protein ECB94_25375	-
A0A3G4VJR7	DT07/RW01	*ECB94_23525*	-	Alpha-amylase	-
A0A3G4VKZ1	DT07/RW01	*ECB94_2372*	-	Porin	*nmpC*

Notes: Gene names correspond to predicted proteins in the proteome; gene ID represents the name of the coding gene in the genome.

**Table 2:** Differential presence of 7 ECP protein-encoding genes in the DT02 genome compared to DT07 and RW01 genomes.

Protein ID	Gene Name	Gene ID	Protein Function	VFDB ID
A0A2S9ZJJ4 *	*tcdA*	-	tRNA cyclic N6-threonylcarbamoyladenosine (37) synthase TcdA	*qbsC*
A0A3G4VIK3	*ECB94_25465*	-	Endonuclease/exonuclease/phosphatase family protein	-
A0A241TCY2	*C9980_10675*	-	Antibiotic biosynthesis monooxygenase	-
A0A2C9PE00	*infC*	*infC*	Translation initiation factor IF-3	-
A0A2S9ZU94	*C9980_13450*	-	DUF2132 domain-containing protein	-
A0A3G4VGP7	*ECB94_21925*	-	Carbohydrate porin	-
A0A3G4VH03	*katG*	-	Catalase-peroxidase	*katG*

Notes: *, only present in RW01; gene name corresponds to predicted proteins in the proteome; gene ID represents the name of coding gene in genome.

**Table 3:** Information for the 15 out of 127 ECP proteins specific to the 1 low-virulence strain with different coding genes in the all 3 strains genomes.

Protein ID	Carrier Strain	Gene Name	Gene ID	Protein Function
A0A241T9V0	DT02	*ECB94_16565*	-	Thioredoxin peroxidase
A0A241TAL0	DT02	*ECB94_16580*	-	Flavodoxin family protein
A0A2C9P9E1	DT07/RW01	*C9980_24925*	-	DUF2492 family protein
A0A2C9PBH5	DT07/RW01	*rpmA*	*rpmA*	50S ribosomal protein
A0A2S9ZM41	DT02	*ECB94_19840*	-	TRAP transporter substrate-binding protein
A0A3G4V6C7	DT07/RW01	*ECB94_03025*	-	OmpA family protein
A0A3G4V8Z6	DT07/RW01	*argH*	-	Argininosuccinate lyase
A0A3G4VAL7	DT07/RW01	*ECB94_06050*	-	Lytic transglycosylase
A0A3G4VBK1	DT07/RW01	*ECB94_12780*	-	DUF2057 domain-containing protein
A0A3G4VD91	DT02	*ECB94_16550*	-	Aminopeptidase P family protein
A0A3G4VDN8	DT07/RW01	*iolC*	-	5-dehydro-2-deoxygluconokinase
A0A3G4VHY4	DT02	*ECB94_24420*	-	Uncharacterized protein
A0A3G4VII2	DT02	*ECB94_19140*	-	TRAP transporter substrate-binding protein
A0A3G4VNN0	DT02/RW01	*ECB94_24780*	-	Nucleotidyltransferase domain-containing protein
A0A3G4VPA6	DT07	*ECB94_26085*	-	Arylesterase

Notes: Gene name corresponds to predicted proteins in the proteome; gene ID represents the name of the coding gene in the genome.

**Table 4:** Comparison of the relative abundance (percentage) of each KEGG pathway in the 3 sets of *V. mediterranei* ECP proteins.

KEGG A Class	KEGG B Class	1265 Proteins	95 Proteins	127 Proteins
Metabolism	Global and overview maps	70.64%	74.19%	68.57%
	Amino acid metabolism	23.55%	12.90%	8.57%
	Carbohydrate metabolism	23.55%	29.03%	37.14%
	Metabolism of cofactors and vitamins	14.68%	22.58%	11.43%
	Energy metabolism	12.33%	6.45%	17.14%
	Nucleotide metabolism	9.83%	3.23%	0
	Metabolism of other amino acids	6.23%	9.68%	2.86%
	Lipid metabolism	5.40%	6.45%	2.86%
	Biosynthesis of other secondary metabolites	4.02%5	3.23%	0
	Glycan biosynthesis and metabolism	3.74%	0	0
	Xenobiotics biodegradation and metabolism	3.74%	3.24%	0
	Metabolism of terpenoids and polyketides	2.35%	0	0
Genetic Information Processing	Translation	9.70%	3.23%	0
	Folding, sorting and degradation	3.19%	6.45%	0
	Replication and repair	2.35%	3.23%	0
	Transcription	0.55%	0	0
Environmental Information Processing	Membrane transport	7.76%	16.13%	14.29%
	Signal transduction	5.54%	0	14.29%
Cellular Processes	Cellular community—prokaryotes	6.37%	12.90%	5.71%
	Cell motility	3.19%	0	11.43%
	Cell growth and death	0.83%	0	0
Organismal Systems	Environmental adaptation	0.97%	0	0
	Immune system	0.69%	0	0

## Data Availability

Data are contained within the article and Supplementary Materials.

## Supplementary Materials

The following supporting information can be downloaded at: https://www.mdpi.com/article/10.3390/ani14050692/s1, Table S1: Information about the 100 ECP proteins without coding genes for the 1265 ECP proteins shared by all 3 strains. Table S2: Information about the 22 ECP proteins without coding genes in the 95 ECP proteins specific to the 2 high-virulence strains. Table S3: Information about the 28 ECP proteins without coding genes in the 127 ECP proteins specific to the 1 low-virulence strain. Table S4: Virulence factors in the 1265 ECP proteins shared by all 3 bacterial strains and their classification in VFDB. Table S5: Virulence factors in the 95 ECP proteins specific to the 2 high-virulence strains and their classification in VFDB. Table S6: Virulence factors in the 127 ECP proteins specific to the low-virulence strain and their classification in VFDB. Table S7: Virulence factors in the 1265 ECP proteins shared by all 3 bacterial strains and their classification in PHI-base. Table S8: Virulence factors in the 95 ECP proteins specific to the 2 high-virulence strains and their classification in PHI-base. Table S9: Virulence factors in the 127 ECP proteins specific to the low-virulence strain and their classification in PHI-base.
