# Supplementary material for: Genomic and Proteomic Analyses of Extracellular Products Reveal Major Virulence Factors Likely Accounting for Differences in Pathogenicity to Bivalves between Vibrio mediterranei Strains
Source: Animals (Basel). 2024 Feb 22;14(5):692. doi: 10.3390/ani14050692 (PMC10930458; doi:10.3390/ani14050692)
Supplement: Supplementary file 1 [file animals-14-00692-s001.zip › Supplementary data/Supplementary data 1.docx]

**Table S1.** Information about the 100 ECP proteins without coding genes for the 1265 ECP proteins shared by all 3 strains.

| **Protein ID** | **Gene name** | **Protein function** |
| --- | --- | --- |
| A0A241T8T2 | *C998_46* | AsnC family transcriptional regulator |
| A0A241T968 | *ECB94_2475* | L-ribulose-5-phosphate 3-epimerase |
| A0A241T9P3 | *C998_1795* | PTS lactose transporter subunit IIB |
| A0A241TB04 | *C998_125* | YibL family ribosome-associated protein |
| A0A241TBG9 | *C998_817* | RNA-dependent RNA-polymerase |
| A0A241TBN2 | *C998_884* | Uncharacterized protein |
| A0A241TCD5 | *gcvH* | Glycine cleavage system H protein |
| A0A2C9P6X7 | *C998_15515* | DUF2498 domain-containing protein |
| A0A2C9P7A5 | *sixA* | Phosphohistidine phosphatase SixA |
| A0A2C9P800 | *glnB* | Nitrogen regulatory protein P-II |
| A0A2C9P874 | *iscA* | Iron-binding protein IscA |
| A0A2C9P8V8 | *C998_1996* | DNA-binding protein |
| A0A2C9P997 | *coaA* | Pantothenate kinase |
| A0A2C9P9E4 | *C998_2454* | ParA family protein |
| A0A2C9P9K1 | *C998_22195* | Multidrug DMT transporter permease |
| A0A2C9PA01 | *tusA* | Sulfur carrier protein TusA |
| A0A2C9PA03 | *C998_23255* | Cytochrome c4 |
| A0A2C9PA79 | *rpsM* | 3S ribosomal protein S13 |
| A0A2C9PA84 | *tatA* | Sec-independent protein translocase protein TatA |
| A0A2C9PAC4 | *rpsI* | 3S ribosomal protein S9 |
| A0A2C9PAN4 | *rpmC* | 5S ribosomal protein L29 |
| A0A2C9PB30 | *C998_752* | P-II family nitrogen regulator |
| A0A2C9PB35 | *rpmB* | 5S ribosomal protein L28 |
| A0A2C9PB36 | *rplK* | 5S ribosomal protein L11 |
| A0A2C9PB48 | *C998_2184* | 5-carboxymethyl-2-hydroxymuconate Delta-isomerase |
| A0A2C9PB58 | *secB* | Protein-export protein SecB |
| A0A2C9PBA5 | *rplP* | 5S ribosomal protein L16 |
| A0A2C9PBQ5 | *rpsU* | 3S ribosomal protein S21 |
| A0A2C9PBQ8 | *minE* | Cell division topological specificity factor |
| A0A2C9PC58 | *ECB94_1225* | N-acetyltransferase |
| A0A2C9PC69 | *hfq* | RNA-binding protein Hfq |
| A0A2C9PC94 | *yceD* | 23S rRNA accumulation protein YceD |
| A0A2C9PCA8 | *C998_5255* | HU family DNA-binding protein |
| A0A2C9PCV7 | *acpP* | Acyl carrier protein |
| A0A2C9PDA6 | *C998_13445* | RNA-binding protein |
| A0A2C9PDE1 | *ECB94_12635* | Zn-ribbon-containing protein |
| A0A2C9PDK1 | *rplT* | 5S ribosomal protein L2 OS=Vibrio mediterranei |
| A0A2C9PE12 | *ihfA* | Integration host factor subunit alpha |
| A0A2C9PEL0 | *ECB94_635* | GntR family transcriptional regulator |
| A0A2C9PEZ9 | *acpP* | Acyl carrier protein |
| A0A2C9PF33 | *C998_2185* | Uncharacterized protein |
| A0A2S9ZGI5 | *C998_14765* | Transcriptional initiation protein Tat |
| A0A2S9ZJ51 | *C998_2357* | DUF2799 domain-containing protein |
| A0A2S9ZJ74 | *rpsD* | 3S ribosomal protein S4 |
| A0A2S9ZMZ4 | *C998_1725* | TIGR153 family protein |
| A0A2S9ZN80 | *bamD* | Outer membrane protein assembly factor BamD |
| A0A2S9ZNA1 | *rplI* | 5S ribosomal protein L9 |
| A0A2S9ZNC0 | *yjjX* | Inosine/xanthosine triphosphatase |
| A0A2S9ZNG5 | *rraA* | Regulator of ribonuclease activity A |
| A0A2S9ZPR5 | *dxs* | 1-deoxy-D-xylulose-5-phosphate synthase |
| A0A2S9ZQC3 | *torD* | Chaperone protein TorD |
| A0A2S9ZRC4 | *gloA* | Aldoketomutase |
| A0A2S9ZRK0 | *lpxD* | UDP-3-O-acylglucosamine N-acyltransferase |
| A0A3G4V6G5 | *mukE* | Chromosome partition protein MukE |
| A0A3G4V6N4 | *moaD* | Molybdopterin synthase sulfur carrier subunit |
| A0A3G4V6V1 | *ECB94_4* | SDR family oxidoreductase |
| A0A3G4V788 | *mukF* | Chromosome partition protein MukF |
| A0A3G4V7J7 | *ECB94_478* | Sugar isomerase domain-containing protein |
| A0A3G4V868 | *ECB94_6445* | Patatin-like phospholipase family protein |
| A0A3G4V8R6 | *dkgB* | 2,5-didehydrogluconate reductase DkgB |
| A0A3G4V8R8 | *cysH* | Phosphoadenosine 5'-phosphosulfate reductase |
| A0A3G4V8S8 | *ECB94_777* | Bifunctional aspartokinase/homoserine dehydrogenase |
| A0A3G4V8V8 | *ECB94_752* | YjbQ family protein |
| A0A3G4V8X8 | *arsC* | Arsenate reductase |
| A0A3G4V981 | *ECB94_3315* | UPF434 protein ECB94_3315 |
| A0A3G4V988 | *thiS* | Sulfur carrier protein ThiS |
| A0A3G4V9Y2 | *tpm* | Thiopurine S-methyltransferase |
| A0A3G4V9Z7 | *ntrC* | DNA-binding transcriptional regulator NtrC |
| A0A3G4VA05 | *ECB94_9675* | GNAT family N-acetyltransferase |
| A0A3G4VA87 | *cpxP* | CpxP family protein OS=Vibrio mediterranei |
| A0A3G4VAB5 | *ECB94_1785* | Autotransporter assembly factor TamA |
| A0A3G4VAF5 | *ECB94_199* | Nicotinamide-nucleotide amidase |
| A0A3G4VAW4 | *rpoS* | RNA polymerase sigma factor RpoS |
| A0A3G4VBS9 | *ECB94_195* | RidA family protein |
| A0A3G4VBY1 | *ECB94_1495* | DUF456 domain-containing protein |
| A0A3G4VC42 | *tmk* | Thymidylate kinase |
| A0A3G4VCB0 | *ECB94_14825* | DNA-binding protein |
| A0A3G4VCL8 | *ECB94_12385* | AMP-dependent synthetase |
| A0A3G4VCQ7 | *ECB94_13415* | Flagellar motor switch protein FliN |
| A0A3G4VD00 | *ECB94_1313* | P-II family nitrogen regulator |
| A0A3G4VED7 | *ECB94_1791* | EIII-Fru |
| A0A3G4VEN2 | *ECB94_9295* | ABC transporter ATP-binding protein |
| A0A3G4VF68 | *ECB94_3155* | DUF257 domain-containing protein |
| A0A3G4VG37 | *infB* | Translation initiation factor IF-2 |
| A0A3G4VGF7 | *ECB94_16285* | SAM-dependent methyltransferase |
| A0A3G4VH62 | *ECB94_1751* | TerB family tellurite resistance protein |
| A0A3G4VH89 | *ECB94_2325* | Dihydrofolate reductase |
| A0A3G4VHD6 | *sfsA* | Sugar fermentation stimulation protein homolog |
| A0A3G4VHH1 | *ECB94_235* | Ysc84 domain-containing protein |
| A0A3G4VHP7 | *ECB94_1511* | YecA family protein |
| A0A3G4VHQ1 | *ECB94_2424* | Outer membrane lipoprotein-sorting protein |
| A0A3G4VI51 | *ECB94_2479* | GFA family protein |
| A0A3G4VIC9 | *ECB94_25145* | Enoyl-CoA hydratase/isomerase family protein |
| A0A3G4VIE3 | *modA* | Molybdate ABC transporter substrate-binding protein |
| A0A3G4VIJ8 | *ECB94_212* | 1-pyrroline-5-carboxylate dehydrogenase |
| A0A3G4VIM9 | *ECB94_259* | N(4)-acetylcytidine amidohydrolase |
| A0A3G4VJ95 | *ECB94_2161* | DNA-binding protein VF53 |
| A0A3G4VK01 | *ECB94_2514* | Enoyl-CoA hydratase |
| A0A3G4VK87 | *ECB94_2557* | DUF2861 family protein |
| A0A3G4VMQ2 | *elbB* | Glyoxalase |

Notes: Gene names correspond to predicted proteins in the proteome.

**Table S2.** Information about the 22 ECP proteins without coding genes in the 95 ECP proteins specific to the 2 high-virulence strains.

| **Protein ID** | **Gene name** | **Protein function** |
| --- | --- | --- |
| A0A2C9P718 | *ccmE* | Cytochrome c-type biogenesis protein CcmE |
| A0A2C9P8G0 | *yajC* | Sec translocon accessory complex subunit YajC |
| A0A2C9PAT8 | *rpsK* | 30S ribosomal protein S11 |
| A0A2S9ZKR7 | *nhaB* | Na(+)/H(+) antiporter NhaB |
| A0A3G4V7B7 | *ECB94_04365* | Cytochrome c-type biogenesis protein |
| A0A3G4V7Q5 | *cobO* | Corrinoid adenosyltransferase |
| A0A3G4V809 | *ECB94_06140* | Gfo/Idh/MocA family oxidoreductase |
| A0A3G4V8N1 | *ECB94_07495* | Uncharacterized protein |
| A0A3G4VA21 | *ECB94_03540* | HAD family hydrolase |
| A0A3G4VAC0 | *mlaD* | Outer membrane lipid asymmetry maintenance protein MlaD |
| A0A3G4VCB7 | *ECB94_14680* | tRNA isopentenyl-2-thiomethyl-A-37 hydroxylase MiaE |
| A0A3G4VCP5 | *trpD* | Anthranilate phosphoribosyltransferase |
| A0A3G4VCT8 | *ECB94_15485* | N-acetyltransferase |
| A0A3G4VDX1 | *ybeY* | Endoribonuclease YbeY |
| A0A3G4VE05 | *ECB94_08020* | Glycosyltransferase family 2 protein |
| A0A3G4VEP8 | *bla* | Beta-lactamase |
| A0A3G4VFA3 | *ECB94_10705* | GNAT family N-acetyltransferase |
| A0A3G4VFN2 | *ECB94_03690* | Chemotaxis protein |
| A0A3G4VI73 | *ECB94_25130* | CoA-acylating methylmalonate-semialdehyde dehydrogenase |
| A0A3G4VIU5 | *trxC* | Thioredoxin TrxC |
| A0A3G4VJX5 | *ECB94_21740* | Uncharacterized protein |
| A0A3G4VP93 | *ECB94_25980* | Agglutination protein |

**Table S3.** Information about the 28 ECP proteins without coding genes in the 127 ECP proteins specific to the 1 low-virulence strain.

| **Protein ID** | **Gene name** | **Protein function** |
| --- | --- | --- |
| A0A241TAI0 | *hcp* | Hydroxylamine reductase |
| A0A2C9PAH8 | *C9980_07650* | FAD assembly factor SdhE |
| A0A2C9PAS6 | *acpS* | Holo-[acyl-carrier-protein] synthase |
| A0A2C9PBN9 | *C9980_07525* | YacL family protein |
| A0A2C9PEQ1 | *C9980_14275* | GNAT family N-acetyltransferase |
| A0A2S9ZGE1 | *cobB* | NAD-dependent protein deacylase |
| A0A2S9ZL83 | *ECB94_17000* | GNAT family N-acetyltransferase |
| A0A3G4V5H3 | *tssI* | Type VI secretion system tip protein VgrG |
| A0A3G4V5N0 | *ECB94_01285* | LysR family transcriptional regulator |
| A0A3G4V5W7 | *ECB94_02095* | Acyl-CoA thioesterase |
| A0A3G4V6Z2 | *ECB94_01600* | Iron-containing alcohol dehydrogenase |
| A0A3G4V708 | *ECB94_02760* | Dimethyl sulfoxide reductase subunit A |
| A0A3G4V7P9 | *ECB94_05340* | NAD(P)H:quinone oxidoreductase |
| A0A3G4V8D7 | *rsmC* | Ribosomal RNA small subunit methyltransferase C |
| A0A3G4VAU4 | *ECB94_09110* | Cytochrome c4 |
| A0A3G4VAW0 | *ECB94_05035* | Transcription elongation factor |
| A0A3G4VEV7 | *ECB94_15325* | Histidine kinase |
| A0A3G4VF22 | *iolB* | 5-deoxy-glucuronate isomerase |
| A0A3G4VFD2 | *ECB94_19405* | GntR family transcriptional regulator |
| A0A3G4VFM5 | *ECB94_16855* | ATP-binding protein |
| A0A3G4VGK8 | *ECB94_19620* | MarR family transcriptional regulator |
| A0A3G4VGT3 | *ECB94_19125* | Galactonate dehydratase |
| A0A3G4VHS2 | *ECB94_21765* | Glutathione S-transferase |
| A0A3G4VIW5 | *ECB94_23005* | Fructose-6-phosphate aldolase |
| A0A3G4VIY7 | *ECB94_26490* | Sulfolactaldehyde 3-reductase |
| A0A3G4VJ09 | *ECB94_21995* | Crp/Fnr family transcriptional regulator |
| A0A3G4VJS0 | *ECB94_17900* | Endoglucanase |
| A0A3G4VK70 | *ECB94_26500* | DeoR/GlpR transcriptional regulator |

**Table S5.** Virulence factors in the 95 ECP proteins specific to the 2 high-virulence strains and their classification in VFDB.

| **Classifications** | **VFDB ID** | **Protein ID** | **Gene ID** | **CDS** |
| --- | --- | --- | --- | --- |
| Adherence | *ompA* | A0A3G4VIN8 | - | OmpA-OmpF porin, OOP family |
|  | *slrA* | A0A3G4VCS1 | - | peptidyl-prolyl cis-trans isomerase B (cyclophilin B) |
| Effector delivery system | *epsG* | A0A2C9PAX7 | - | general secretion pathway protein G |
|  | *vtrA* | A0A3G4V8V7 | - | cholera toxin transcriptional activator |
|  | *virB* | A0A3G4VG68 | - | chromosome partitioning protein, ParB family |
| Immune modulation | *gmhA* | A0A2C9P8W5 | - | DnaA initiator-associating protein |
|  | *kdtB* | A0A3G4V8X6 | *coaD* | coaD |
|  | *fadD13* | A0A3G4VBN3 |  | long-chain acyl-CoA synthetase |
| Antimicrobial activity/  Competitive advantage | *mtrC* | A0A3G4V9P3 | - | membrane fusion protein, multidrug efflux system |
| Nutritional/Metabolic factor | *qbsC* | A0A2S9ZJJ4* | - | HesA/MoeB/ThiF family protein |
|  | *hemG* | A0A3G4V9I0 | - | menaquinone-dependent protoporphyrinogen oxidase |
|  | *pvdY* | A0A3G4VHM8 | - | acetyl CoA:N6-hydroxylysine acetyl transferase |
| Stress survival | *katG* | A0A3G4VH03 | - | catalase-peroxidase |

Notes: *, only present in RW01.

**Table S6.** Virulence factors in the 127 ECP proteins specific to the low-virulence strain and their classification in VFDB.

| **Classifications** | **VFDB ID** | **Protein ID** | **Gene ID** | **CDS** |
| --- | --- | --- | --- | --- |
| Adherence | *aaa* | A0A3G4VE27 | - | N-acetylmuramoyl-L-alanine amidase |
| Biofilm | *flgI* | A0A2C9PCE5 | - | flagellar P-ring protein precursor FlgI |
| Effector delivery system | *tssA1* | A0A3G4V5G4 | - | type VI secretion system protein ImpA |
|  | *btrS* | A0A3G4V7G9 | - | RNA polymerase sigma-70 factor, ECF subfamily |
| Immune modulation | *nuoG* | A0A3G4VBT7 | - | formate dehydrogenase major subunit |
| Motility | *cheY* | A0A2S9ZTW6 | - | two-component system, chemotaxis family, response regulator CheY |
|  | *flgT* | A0A3G4V781 | - | flagellar basal-body protein |
|  | *fliK* | A0A3G4VAK1 | - | flagellar hook-length control protein FliK |
| Nutritional/Metabolic factor | *narH* | A0A3G4V6I2 | - | tetrathionate reductase subunit B |
|  | *DDA3937_RS14700* | A0A3G4V895 | - | iron complex outermembrane recepter protein |
|  | *hemM* | A0A3G4VBF8 | - | outer membrane lipoprotein LolB |
|  | *ASA_RS16545* | A0A3G4VC15 | - | putative lipoprotein |
|  | *dhbF* | A0A3G4VFD3 | *prpE* | PrpE |
|  | *mbtN* | A0A3G4VKL0 | - | acyl-CoA dehydrogenase |
| Regulation | *mprA* | A0A3G4VEG6 | - | phosphoserine phosphatase RsbU/P |

**Table S8.** Virulence factors in the 95 ECP proteins specific to the 2 high-virulence stains and their classification in PHI-base.

| **Classifications** | **PHI ID** | **Protein ID** | **Gene ID** | **CDS** |
| --- | --- | --- | --- | --- |
| Increased virulence(hypervirulence) | *Oma1* | A0A3G4VM64 | - | Zn-dependent protease |
| Loss of pathogenicity | *MoIlv2* | A0A3G4VD95 | - | tartronate-semialdehyde synthase |
|  | *GLX3* | A0A3G4VIZ8 | - | putative intracellular protease/amidase |
| Reduced virulence | *PdhR* | A0A2C9P8E1 | *pdhR* | pdhR |
|  | *TatB* | A0A2C9PAG5 | - | sec-independent protein translocase protein TatB |
|  | *PilA* | A0A2C9PAX7 | - | general secretion pathway protein G |
|  | *Uba2* | A0A2S9ZJJ4* | - | HesA/MoeB/ThiF family protein |
|  | *LeuO* | A0A2S9ZRL9 | - | transcriptional regulator |
|  | *PpiB* | A0A3G4VCS1 | - | peptidyl-prolyl cis-trans isomerase B (cyclophilin B) |
|  | *AccD* | A0A3G4VD41 | - | acetyl-CoA carboxylase carboxyl transferase subunit beta |
|  | *RED1* | A0A3G4VEY8 | - | uncharacterized protein |
|  | *OmpA* | A0A3G4VIN8 | - | OmpA-OmpF porin, OOP family |
|  | *PG0343* | A0A3G4VM74 | - | Cys/Met metabolism pyridoxal-phosphate-dependentenzyme |
| Reduced virulence/Unaffected pathogenicity | *Rv3114* | A0A3G4V7R0 | - | tRNA(adenine34) deaminase |
|  | *FadD* | A0A3G4VBN3 | - | long-chain acyl-CoA synthetase |
|  | *MakatG1* | A0A3G4VH03 | - | catalase-peroxidase |
|  | *VpDsbA1(VP3054)* | A0A3G4VKI7 | - | thiol-disulfide isomerase and thioredoxin |
| Unaffected pathogenicity | *ToxR* | A0A3G4V8V7 | - | cholera toxin transcriptional activator |
|  | *MexA* | A0A3G4V9P3 | - | membrane fusion protein, multidrug efflux system |
|  | *CadC* | A0A3G4VJD5 | *hlyU* | hlyU |

Notes: *, only present in RW01.

**Table S9.** Virulence factors in the 127 ECP proteins specific to the low-virulence strain and their classification in PHI-base.

| **Classifications** | **PHI ID** | **Protein ID** | **Gene ID** | **CDS** |
| --- | --- | --- | --- | --- |
| Increased virulence (hypervirulence) | *CjrA* | A0A3G4VC15 | - | putative lipoprotein |
|  | *Bcas0263* | A0A2S9ZTW6 | - | two-component system, chemotaxis family, response regulator CheY |
| Lethal | *LYS4* | A0A3G4VDQ9 | - | aconitate hydratase |
| Loss of pathogenicity | *MoARG5_6* | A0A3G4V8S4 | *argC* | argC |
|  | *MoSCAD2_(MGG_08690)* | A0A3G4VKL0 | - | acyl-CoA dehydrogenase |
| Reduced virulence | *AmiC* | A0A3G4VE27 | - | N-acetylmuramoyl-L-alanine amidase |
|  | *PtsN* | A0A2C9PAT5 | - | PTS system, nitrogen regulatory IIA component |
|  | *PtsI* | A0A3G4VB00 | - | phosphotransferase system, enzyme I, PtsP |
|  | *NapA* | A0A3G4VBT7 | - | formate dehydrogenase major subunit |
|  | *BioDA_(ALFA_049090)* | A0A3G4VFJ6 | - | dethiobiotin synthetase |
|  | *PotD* | A0A3G4VE49 | - | putative spermidine/putrescine transport system substrate-binding protein |
|  | *XC_3287* | A0A3G4VE68 | - | alpha-ribazole phosphatase |
|  | *CovR* | A0A3G4VEG6 | - | phosphoserine phosphatase RsbU/P |
|  | *Acs* | A0A3G4VFD3 | *prpE* | prpE |
|  | *PqsD* | A0A3G4V629 | - | 3-Oxoacyl-(acyl-carrier-protein (ACP)) synthase III domain protein |
|  | *PqsR* | A0A2C9P7G5 | - | LysR family transcriptional regulator |
|  | *BipA* | A0A3G4VBM8 | - | elongation factor G |
|  | *AspC_(PXO_02420)* | A0A3G4V7K7 | - | cystathione beta-lyase |
|  | *FiuA* | A0A3G4V895 | - | iron complex outermembrane recepter protein |
|  | *PXO_03326* | A0A3G4VF66 | - | hypothetical protein |
|  | *AldA_(PSPTO_0092)* | A0A3G4VH78 | - | aldehyde dehydrogenase |
|  | *TreS* | A0A3G4VHC8 | - | amylosucrase |
|  | *ChiB* | A0A3G4VJ10 | *chitinase* | chitinase |
|  | *Rhr2* | A0A3G4VIA8 | - | CbbY family protein |
|  | *LeuO* | A0A3G4VM21 | - | putative Transcriptional regulator, LysR family |
|  | *MalS* | A0A3G4VKZ8 | - | alpha-amylase |
|  | *PstS* | A0A3G4VIU1 | - | phosphate transport system substrate-binding protein |
|  | *Vsp* | A0A3G4VGR5 | - | SI family secreted trypsin-like serine protease |
|  | *LeuO* | A0A3G4VM96 | - | transcriptional regulator, LysR family |
| Reduced virulence/  Unaffected pathogenicity | *VpDsbA2_(VPA1271)* | A0A3G4VBP6 | - | thiol:disulfide interchange protein DsbA |
| Unaffected pathogenicity | *Trr1* | A0A3G4VC09 | - | glutamate synthase (NADPH/NADH) small chain |
|  | *XC_1273* | A0A3G4VES8 | - | voltage-gated potassium channel |
|  | *TtrB* | A0A3G4V6I2 | - | tetrathionate reductase subunit B |
|  | *Ecf11* | A0A3G4V7G9 | - | RNA polymerase sigma-70 factor, ECF subfamily |
|  | *OdhB* | A0A3G4VNX0 | - | pyruvate dehydrogenase E2 component (dihydrolipoamide acetyltransferase) |
